# Quality of Life Evaluation

**DOI:** 10.5812/kowsar.22287523.3548

**Published:** 2012-01-01

**Authors:** Laxmaiah Manchikanti

**Affiliations:** 1 Pain Management Center of Paducah, Paducah, USA

**Keywords:** Quality of Life, Discectomy, Surgery

Dear Editor,

Farzanegan et al. described quality of life evaluations of patients undergoing lumbar discectomy using the short-form 36 (SF-36) (1). They provided physical and mental health scores of patients which improved significantly 6 and 12 months after lumbar discectomy, leading to the conclusion that lumbar discectomy improves both the physical and mental health subscale of the quality of life in patients with chronic disc herniation. 

Quality of life and functional status improvement is evaluated by many available tests; one of them SF-36 as utilized in this study. While all these instruments are considered as objective evaluations, they all depend on subjective information. SF-36 is a generic measure, as opposed to one that targets a specific age, disease, or treatment group. Thus, for low back pain, either the Oswestry Disability Index (ODI) (2) or Roland Morris Disability Questionnaire (RDQ) (3) are considered as more specific measures. Even so, the studies of validity of SF-36 from many types of research have yielded content, concurrent, criterion, construct, and predictive evidence of validity (4). Consequently, the SF-36 has been shown to be sensitive to change (5, 6) and able to differentiate between treatment responders and non-responders (7, 8). Further, SF-36 has been used as a validation tool in the development of new disease-specific instruments (9, 10), including a pain-specific tool (10, 11).

However, there has not been any significant description of a clinically significant change in any condition for SF-36. In contrast, clinically significant change has been described as a 15-point change in patients who undergo spinal fusion before surgery and at follow-up for ODI (4). Others have described a change of 4 points as the minimum difference in mean scores between the group that carried clinical significance. On the same grounds, it has been suggested that the smallest change likely to be clinically significant for RDQ lies between 2.5 and 5 points (4). In recent years, it has been stated that a 40% or 50% change from pre-treatment level as the appropriate change (12, 13).

Consequently, when these issues are taken into consideration, do the changes described in Farzanegan et al.’s manuscript (1), which looked rather dramatic with significantly high P values, really indicate clinical significance? Essentially the changes described in this manuscript for physical health scores, which are the best, show only 18% improvement, whereas for mental health, it appears that they may be less than 15% from 38.16 to 43.48 (value for 12 months appears to be wrong in Figure 2). 

The clinical implications of the study include not only its usefulness in evaluating lumbar discectomy, but also the questions about meaningful change, which is clinically significant. In the future, studies such as this one should be compared with other disease specific tests such as RDQ. In this case, most patients had subacute pain, whereas ODI is for patients with chronic pain.
